# Genomic Prediction and Genetic Correlation of Agronomic, Blackleg Disease, and Seed Quality Traits in Canola (*Brassica napus* L.)

**DOI:** 10.3390/plants9060719

**Published:** 2020-06-05

**Authors:** Mulusew Fikere, Denise M. Barbulescu, M. Michelle Malmberg, Pankaj Maharjan, Phillip A. Salisbury, Surya Kant, Joe Panozzo, Sally Norton, German C. Spangenberg, Noel O. I. Cogan, Hans D. Daetwyler

**Affiliations:** 1 School of Applied Systems Biology, La Trobe University, Bundoora, VIC 3086, Australia; mulusew.ali@agriculture.vic.gov.au (M.F.); Michelle.malmberg@agriculture.vic.gov.au (M.M.M.); german.spangenberg@agriculture.vic.gov.au (G.C.S.); noel.cogan@agriculture.vic.gov.au (N.O.I.C.); 2 Agriculture Victoria, AgriBio, Centre for AgriBioscience, Bundoora, VIC 3083, Australia; p.salisbury@bigpond.com; 3 Queensland Alliance for Agriculture and Food Innovation (QAAFI), The University of Queensland, Brisbane, QLD 4072, Australia; 4 Agriculture Victoria, Grains Innovation Park, Horsham, VIC 3400, Australia; denise.barbulescu@agriculture.vic.gov.au (D.M.B.); pankaj.maharjan@agriculture.vic.gov.au (P.M.); surya.kant@agriculture.vic.gov.au (S.K.); joe.panozzo@agriculture.vic.gov.au (J.P.); sally.norton@agriculture.vic.gov.au (S.N.); 5 Faculty of Veterinary and Agricultural Sciences, The University of Melbourne, Parkville, VIC 3010, Australia; 6 Centre for Agricultural Innovation, The University of Melbourne, Parkville, VIC 3010, Australia

**Keywords:** canola, fatty acid, blackleg disease, genotype-by-environment interaction, genomic selection, genetic correlation, grain yield

## Abstract

Genomic selection accelerates genetic progress in crop breeding through the prediction of future phenotypes of selection candidates based on only their genomic information. Here we report genetic correlations and genomic prediction accuracies in 22 agronomic, disease, and seed quality traits measured across multiple years (2015–2017) in replicated trials under rain-fed and irrigated conditions in Victoria, Australia. Two hundred and two spring canola lines were genotyped for 62,082 Single Nucleotide Polymorphisms (SNPs) using transcriptomic genotype-by-sequencing (GBSt). Traits were evaluated in single trait and bivariate genomic best linear unbiased prediction (GBLUP) models and cross-validation. GBLUP were also expanded to include genotype-by-environment G × E interactions. Genomic heritability varied from 0.31to 0.66. Genetic correlations were highly positive within traits across locations and years. Oil content was positively correlated with most agronomic traits. Strong, not previously documented, negative correlations were observed between average internal infection (a measure of blackleg disease) and arachidic and stearic acids. The genetic correlations between fatty acid traits followed the expected patterns based on oil biosynthesis pathways. Genomic prediction accuracy ranged from 0.29 for emergence count to 0.69 for seed yield. The incorporation of G × E translates into improved prediction accuracy by up to 6%. The genomic prediction accuracies achieved indicate that genomic selection is ready for application in canola breeding.

## 1. Introduction

Canola (*Brassica napus* L., AACC, 2n = 38) is the second most important oilseed worldwide, after soybean [1]. Global food production has to double by 2050 to feed the growing human population [2], which underscores the importance of accelerating crop improvement. The development of molecular plant breeding using high throughput genetic markers has enabled breeders to accelerate genetic gain and improved several agronomic and quality traits. In addition, the recent development of advanced phenotypic data collection using drone imaging technology is creating opportunities to obtain high-quality and a real-time records from large experimental sites [3,4].

In canola, high-priority breeding targets are high-yielding cultivars with improved blackleg resistance and improved oil quality. Several studies have shown that canola oil has a beneficial fatty acid (FA) profile for human nutrition, due to its lower saturated FA content. Canola cultivars vary in their FA profiles [5,6] and the presence of such variation allowed breeders to reduce toxic seed components (erucic acid and glucosinolate) to produce double-zero canola oil seed. Similarly, through several genetic introgressions it became possible to increase the oleic acid [C18:1] to 60% in canola seed oil [7,8] and to determine the optimal FA composition for cooking and frying quality as has been determined as 60% oleic acid [C18:1], 4% palmitic acid [C16:0], and 2% stearic acid [C18:0], arachidic acid (20:0), with an optimal 2:1 ratio of linoleic (C18:2) and linolenic acid (C18:3) [8,9]. In addition, several varieties resistant to blackleg disease (caused by *Leptosphaeria maculans*) were developed and have significantly improved canola yield per unit time and space, which was extensively reviewed by Wouw et al., 2016 [10]. These achievements were obtained after decades of long and intensive breeding in multi-environment trials (MET). Most canola traits are complex and have many genes contributing towards genetic variation. A variety of new breeding tools, including genomic selection, are well suited to improve complex traits and could accelerate genetic gain.

Genomic selection (GS) is a new and advanced breeding method that can shorten long breeding cycles and has become a powerful selection tool to increase genetic gain for complex traits [11]. The approach uses a reference population, which is phenotyped and genotyped, to predict the genetic values of selection candidates which are only genotyped. Recent studies in canola [12] demonstrated that GS accuracy is in the usable range for reducing the breeding cycle [13,14,15]. The role of GS in accelerating crop improvement, the factors affecting prediction accuracy, and possible strategies for future application have been reviewed [16,17,18]. Genomics also allow for the estimation of genetic correlations between traits. This information is useful to understand the relative merit of multi-trait GS models [19]. Multi-trait GS have been shown to increase prediction accuracy for low heritability traits which are correlated to a higher heritability trait. Furthermore, genetic correlations are useful when devising selection indices [20].

Several statistical models have been proposed to explore the potential of GS in plant breeding [18,21]. GS methods have been assessed for agronomic, disease, and quality traits and have been shown to have an advantage over phenotypic selection in plants. For instance, Rutski et al., 2012 [22] compared genomic prediction models for fusarium head blight resistance in wheat and found that the marker based prediction model leads to a better accuracy than selection based on phenotypes. Additional studies further demonstrated the advantages of GS for improving genetic gain per unit time over pedigree selection alone [23,24]. The potential role of GS in canola breeding programs and the significance of high-throughput markers were reviewed by Snowdon et al., 2012 [25] concluding that these advanced tools will play an important role in germplasm development in canola.

Published genomic prediction accuracies have varied in canola, depending on traits, population diversity, and the extent of genotype-by-environment (G × E) interaction observed. A study by Wurschum et al. [13] observed a higher genomic prediction accuracy for flowering time and plant height compared to seed quality traits, suggesting that some of quality traits have been under intense selection in the breeding program. Jan et al., 2016 [14] used a canola reference population to evaluate genomic prediction accuracy of several quality and yield-related traits and found a high accuracy for seed oil content (0.81). The potential of GS for seedling emergence and blackleg disease resistance in a set of spring and winter lines were reported in Fikere et al., 2018 [15] demonstrating how GS effectively determines the most resistant lines from the field trial. One of the major factors for improving genomic prediction accuracy is the variability of plant performance across environments. Multi-environment trials are commonly applied to understand G × E interactions of cultivars before release [26,27]. Studies showed that gene effects vary among the same cultivars tested at different environments, which revealed that breeders need to consider this source of variation in the prediction model. Several studies in other crop species have observed that genomic prediction accuracies were increased when breeding values were estimated with a G × E interaction effect in crops other than canola (e.g., [28,29]. However, incorporating G × E interactions into genomic prediction for canola has not been extensively studied.

While genomic prediction accuracy has been investigated for several traits in canola, several knowledge gaps remain. Relatively little is known about genomic prediction performance for detailed FA composition traits and for some agronomic traits in canola (e.g., lodging, shattering, and vigor). In this study, we investigated genomic prediction in 202 spring canola lines for a total of 22 agronomic, disease-resistance and FA traits measured in up to 6 field trials under irrigated and rain-fed conditions for three consecutive years 2015–2017 in Victoria, Australia. Specifically, our aims were to: (i) assess genetic and phenotypic correlations between agronomic, disease, and FA traits (ii) evaluate genomic prediction accuracy for these traits within locations using genomic best linear unbiased prediction (GBLUP) and (iii) incorporate G×E interactions into GBLUP and evaluate their effect on prediction accuracy across locations, years, and watering condition.

## 2. Results

### 2.1. Phenotypic Variation and Trait Heritability

**Figure 1:**
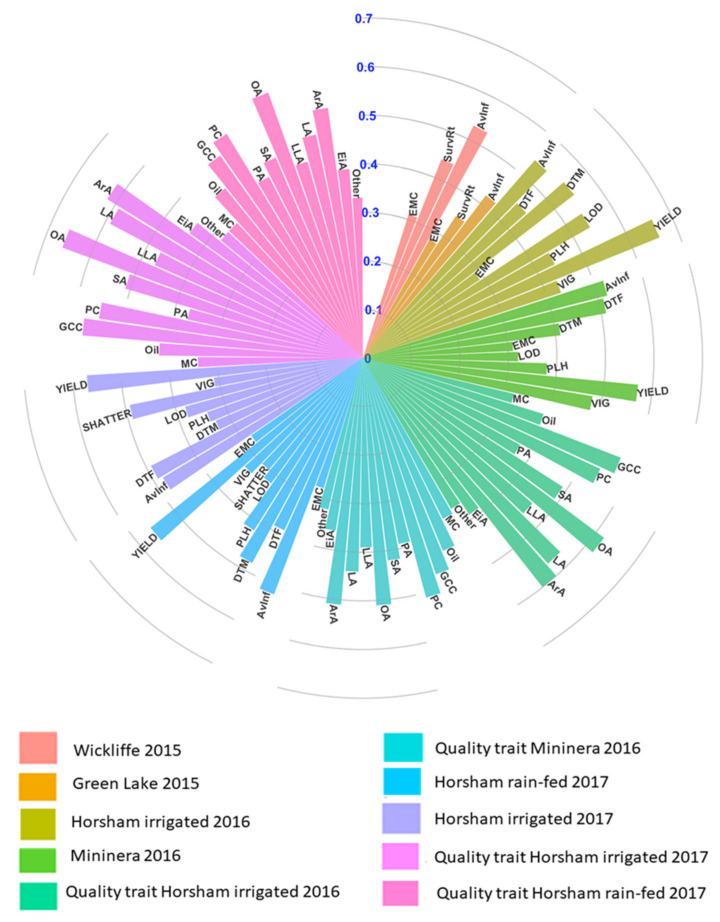
Genomic heritability (h^2^) at Wickliffe 2015 and Green Lake 2015, Horsham irrigated 2016 and 2017, Horsham rain-fed 2017, and Mininera 2016. AvInf = average internal infection, SurvRt = survival rate, DTF = days to flowering, DTM = days to maturity, EMC = emergence score, LOD = lodging score, SHA = shattering, PLH = plant height (cm), YIELD = seed weight per plot (g/plot), VIG = vigor score, MC = moisture content, Oil = oil content, GCC = glucosinolate (μmol/g seed), PC = seed protein content, PA = palmitic acid (C16:0), SA = stearic acid (C18:0), OA = oleic acid (C18:1), LA = linoleic acid (C18:2), LLA = linolenic acid (C18:3), ArA = arachidic acid (C20:0), EiA = eicosenoic acid (20:1), Other.

The phenotypic performance (best linear unbiased estimates (BLUEs)), broad-sense heritability shown in (Table S2a–c) and genomic heritability (Figure 1) for 202 canola lines were recorded under rain-fed sites (Wickliffe, Green Lake, Mininera and Horsham rain-fed) and irrigated conditions (Horsham irrigated 2016 and 2017 sites) during the 2015–2017 growing seasons was determined. The greatest phenotypic variation was observed in average internal infection (AvInf) and seed yield per plot (YIELD) across locations and years. For instance, AvInf ranged from 7.22 at Horsham irrigated 2016 site to 79.65 at Wickliffe. In 2015, higher seedling emergence were observed at Wickliffe than at Green Lake. The highest mean yield (2794.16 g/plot) was recorded at Horsham irrigated 2017 and the lowest yield (1016.64 g/plot) was recorded at Mininera site 2016. In addition, even if they were protected, some of the agronomical traits’ experimental sites showed a disease development trend.

We have investigated variation between quality traits (oil content and FA compositions) under rain-fed (Mininera and Horsham rain-fed) and irrigated (Horsham irrigated) conditions during the 2016 and 2017 growing season. We found that ranges differed in some traits at different sites and in different years. For instance, glucosinolate content (GCC) were 4.92 and 5.04 µmol/g at Mininera (MI16) and Horsham irrigated (HrI16), respectively, whereas in the 2017 trials GCC were recorded 6.97 to 8.50 µmol/g. However, most traits remained in narrow ranges across sites and years, such as seed protein content (PC) was exhibited 20.02% at MI16 and 20.59% at HrI16, linolenic acid (LLA) were 20.52% at MI16 and 19.72% at HrI16, whereas eicosenoic acid [30] = 0.90% at MI16 and 0.98% at HrI16 (Table S2c).

We further estimated H^2^ and h^2^ for all traits across locations (Table S2a–c and Figure 1). The highest H^2^ were achieved for seed yield (0.72) and 0.70 at Horsham irrigated (Hr17) and HrI16 respectively, followed by AvInf (H^2^ = 0.71) at HrI16 and 0.69 at MI16. While the lowest was recorded for emergence count (H^2^ = 0.32) at Green Lake (GL15) and 0.33 at Hr17 Horsham irrigated 2016. Similarly, H^2^ for quality traits ranged from 0.41 for EiA (C20:1) at MI16 and HrI17 to 0.70 for oleic acid (OA, C18:1) at HrI16 and HrI17 (Table S2c). Furthermore, the estimated genomic heritability for agronomic and quality traits follow a similar trend with broad sense heritability (Figure 1). For instance, seed yield and average internal infection consistently remain the highest h^2^ and seedling emergence was generally the lowest h^2^ across trials. In seed composition traits, OA had the highest h^2^ (0.62) followed by glucosinolate content (0.56), and the lowest was recorded for EiA (h^2^ = 0.25) and other (h^2^ = 0.21) uncalibrated FA compositions across watering conditions. Overall, the moderate to high genetic heritability across sites suggests that these traits can be genetically improved and genetic gain can be achieved. A summary of phenotypes along with broad sense heritability is presented in Table S2a–c.

### 2.2. Genomic Data and Population Relatedness

**Figure 2:**
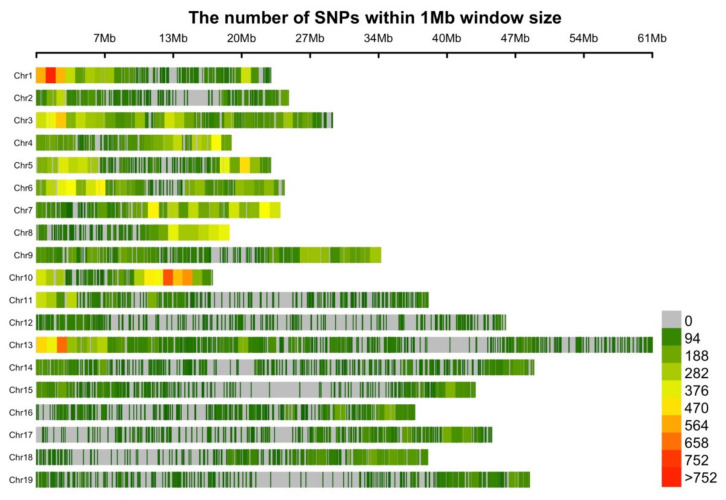
Distribution of single nucleotide polymorphisms SNPs in non-overlapping 1Mb windows for 19 B. napus chromosomes (A Chr 1-10 and C Chr 11-19 sub-genomes).

The single nucleotide polymorphism (SNP) distribution of the 62,082 SNP remaining after imposing a Beagle R-square threshold of 0.5 were plotted in 1MB (mega basepair) windows across the *B. napus* L. genome (Figure 2). Variant distribution was not completely uniform across the chromosomes. We detected an average of 470 SNPs per 1 Mb. The highest SNP density (>752 SNPs/1 Mb) was observed on chromosomes A01, A10 and C03. The lowest average SNP density was found on chromosome C09, C02 and C06 (<94 SNPs/1 Mb). In general, average SNP density was higher on the A than the C sub-genome.

**Figure 3:**
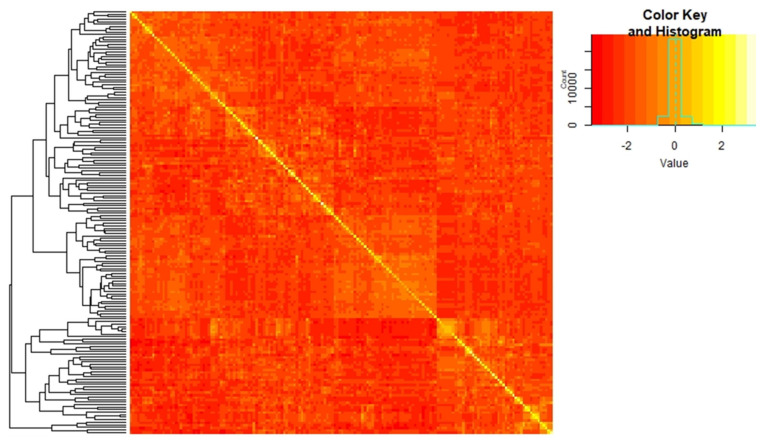
Heat map depicting genomic relationships for 202 canola lines, where yellow represents more and red represents less related individuals.

The genetic relatedness between 202 canola lines genotyped using 62,082 SNP markers is represented as a heat map of the **G** matrix (Figure 3). Small to moderate relatedness between lines were observed. While there were small pockets of higher relatedness (e.g., spring lines with winter background forming a separate cluster, bottom-right of Figure 3), the majority of the lines were not closely related.

### 2.3. Correlations within and between Agronomic and Fatty Acid Traits

Phenotypic correlation coefficients between 10 agronomic and disease as well as 12 quality traits tested under rain-fed and irrigated conditions during the 2015–2017 growing seasons are shown in Figures S2 and S3. A significant positive phenotypic correlation coefficient was observed between the same traits at different sites and years, for instance for days to flowering (DTF) (r = 0.72), AvInf (r = 0.51), plant height (PLH, r = 0.55) and YIELD (r = 0.52). Similarly, some traits followed expected trends such as the strong negative correlations coefficients were observed for AvInf with YIELD, DTF, survival rate (SurvRt) and lodging score (LOD). This confirmed the antagonistic relationship between disease pressure and yield related traits. We observed that when disease pressure was high, the number of lodged plants were consistently high. This confirms that blackleg (*L. maculans*) severely affects canola plants at various growth stages, compromising susceptible cultivars and causing heavy yield loss. Furthermore, strong and positive phenotypic correlations were observed between vigor score (VIG) and emergence score (EMC, r = 0.54), PLH and YIELD (r = 0.55), indicating that taller plants tend to have a higher number of pods per plant and seed per pod. A similar trend was observed by Porta-Puglia et al., 2000 [31] in field pea and found that taller plants had a yield advantages over the dwarf plants due to more pods per node.

**Figure 4:**
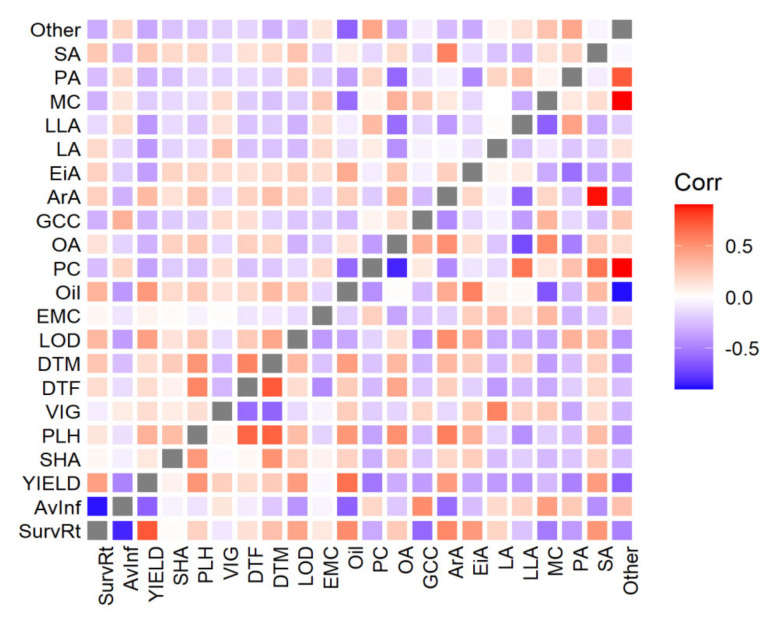
Phenotypic (above diagonal) and genetic (below diagonal) correlations between agronomic, disease, and quality traits. Each square represents the mean correlation across all trials for that trait combination. Abbreviations: AvInf = average internal infection, SurvRt = survival rate, DTF = days to flowering, DTM = days to maturity, EMC = emergence count, LOD = lodging percentage, PLH = plant height, SHA = shattering, YIELD = seed weight per plot, VIG = vigor, MC = moisture content (%), Oil = oil content, GCC = glucosinolate (μmol/g seed), PC = seed protein content, PA (C16:0) = palmitic acid, SA (C18:0) = stearic acid, OA = oleic acid (C18:1), LA = linoleic acid (C18:2), LLA = linolenic acid (C18:3), ArA = arachidic acid (%, C20:0), EiA = eicosenoic acid (C20:1), Other = other seed compounds. Mean, min, and max of genetic correlation SE 0.14, 0.03, 0.27, respectively, and 0.05, 0.01, and 0.08 for phenotypic correlations, respectively.

Genetic correlations were calculated using bivariate GBLUP models. While the trend in genetic correlations was similar to the phenotypic values, genetic correlations generally were greater in magnitude (i.e., more positive or more negative). Genetic correlations (below diagonal) within quality and agronomic traits are shown in Figures S2 and S3. Strong negative correlations were exhibited for AvInf with YIELD and LOD. LLA and OA were strongly negatively correlated (−0.54). Similarly, YIELD was negatively correlated with several FAs (EiA −0.33, linoleic acid (LA) −0.41, LLA −0.39, palmitic acid (PA) −0.40) (Figure 4). A negative correlation was observed for AvInf with most of FA composition traits (e.g., oil content, −0.61), while PLH and SurvRt positively correlated with Oil.

Genetic correlations within FAs and agronomic traits are shown in Figure 4. Oil content was positively correlated to OA and stearic acid (SA). Negative correlations were found between oil with LLA and MC. DTF was positively correlated with Oil, suggesting that late flowering cultivars have more time to accumulate seed oil. We observed that LOD altered the FA composition in canola. The unsaturated FA content (LA, and LLA) decreased, whereas that of saturated FAs (PA, SA, and arachidic acid (ArA)) increased with the increasing LOD (Figure 4). In addition, we observed that AvInf weakly correlated with PA (0.25) and negatively correlated with OA (−0.21), ArA (−0.57), and SA (−0.44). Taken together, the genetic correlations substantiated phenotypic correlations.

### 2.4. Genomic Prediction Accuracy within Site and Year

**Figure 5:**
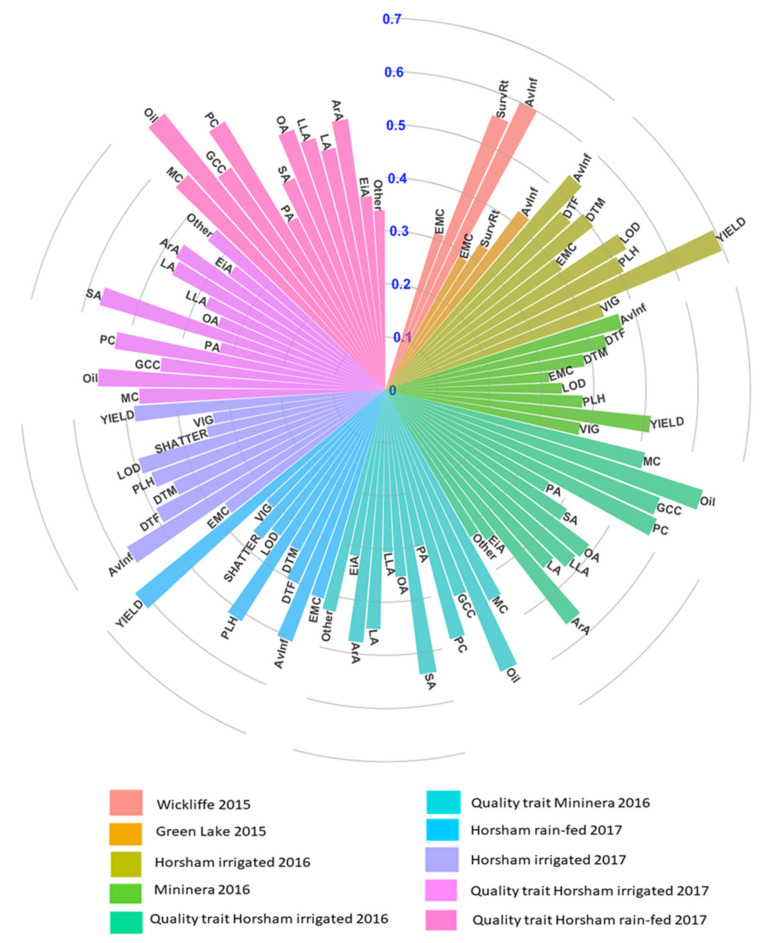
Genomic prediction accuracy for agronomic and quality traits within location. AvInf = Average internal infection, DTF = days to flowering, DTM = days to maturity, EMC = emergence count, LOD = lodging score, PLH = plant height (cm), SWP = seed weight per plot (g/plot), VIG = vigor score, MC = moisture content, Oil = oil content, GCC = glucosinolate (μmol/g seed), PC = seed protein content, PA = palmitic acid (C16:0), SA = stearic acid (C18:0), OA = oleic acid (C18:1), LA = linoleic acid (C18:2), LLA = linolenic acid (C18:3), ArA = arachidic acid (C20:0), EiA = eicosenoic acid (20:1), other.

A total of 202 canola lines genotyped with 62,082 SNP markers were used to evaluate the genomic prediction for agronomic, disease, and quality traits for each site using GBLUP method with cross-validations (Figure 5). The highest prediction accuracy was achieved for YIELD 0.69 and 0.61 at Horsham irrigated 2016 and 2017 respectively. AvInf was the second highest prediction accuracy (0.60 at Wickliffe and 0.58 at Horsham irrigated 2017) followed by SurvRt (0.56) at Wickliffe 2015.

Intermediate prediction accuracy was observed for DTF ranging from 0.4 at Hr17 to 0.49 at HrI17 and 0.5 for days to maturity (DTM) at HrI16. The prediction accuracy for PLH was 0.38 at MI16 to 0.52 at Hr17 and LOD the highest accuracy was 0.53 recorded at HrI16 and the lowest was 0.34 at Hr17 and MI16. Below average (<0.42) prediction accuracy were obtained for VIG and EMC under rain-fed and irrigated conditions.

The highest prediction accuracy for quality traits was recorded for Oil (r = 0.64) followed by ArA (r = 0.58) and PC (r = 0.58) at Horsham irrigated 2016 and 2017, whereas EiA had the lowest (r = 0.31 and 0.34) prediction accuracy at Mininera 2016 and Horsham irrigated 2016, respectively. This trait was also the lowest heritability at the two sites under both watering conditions (Figure 1, Table S2a–c). Overall, for the fatty acid composition traits prediction accuracy trended similarly between rain-fed and irrigated conditions. Furthermore, prediction accuracy under the rain-fed conditions was generally lower than under irrigation. Generally, the genomic prediction accuracy followed a similar trend to genetic heritability (h^2^).

Regressions of BLUEs on genomic estimated breeding values (GEBVs) were calculated to estimate the bias (slope) for all traits within locations and years. A slope of less than 1 or greater than 1 means the GEBV over- or under-predict the phenotype, while the expected slope is 1. The average slope ranged from 0.8 to 2.4 across trials (Table S3a–d) but was generally close to 1, with a trend towards under-prediction of phenotypes.

### 2.5. Environmental Factor Combination in the Models

**Figure 6:**
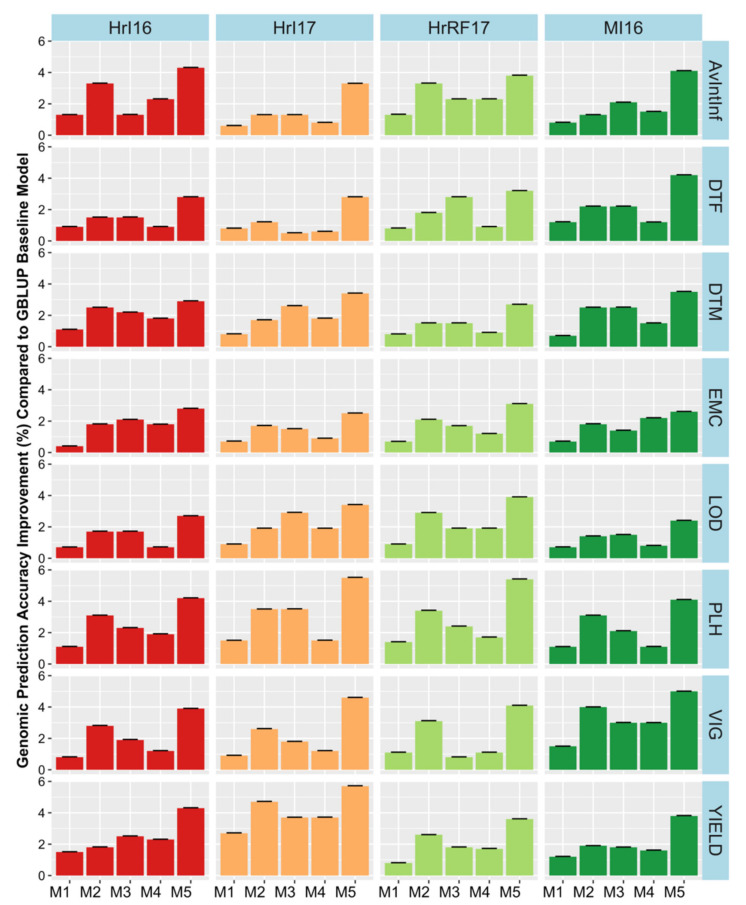
Genomic prediction accuracy improvement for each environmental factor combination across models (M1–M5) compared to the baseline model in field traits measured in agronomic trials. $\mathrm{Accuracy}\;\mathrm{Improvement}\;=(M_{i}-M_{\mathrm{base}\;})\times 100$ where M is prediction accuracy obtained from i environmental factor combination G × E term model and M_base_ is the within site prediction accuracy (Figure 5, Table S3a–d). Models were: M1 = Adding fixed terms such as year, location and water into the genomic best linear unbiased prediction (GBLUP) base model without interactions. In M2, M3 and M4 a random interaction term of genotype by year, location and water condition was added in each model, respectively. Finally, the fifth model M5combined all random interactions. Hr = Horsham, MI = Mininera, AvInf = Average internal infection, DTF = days to flowering, DTM = days to maturity, EMC = emergence count, LOD = lodging score, PLH = plant height (cm), SWP = seed weight per plot (g/plot), VIG = vigor score

**Figure 7:**
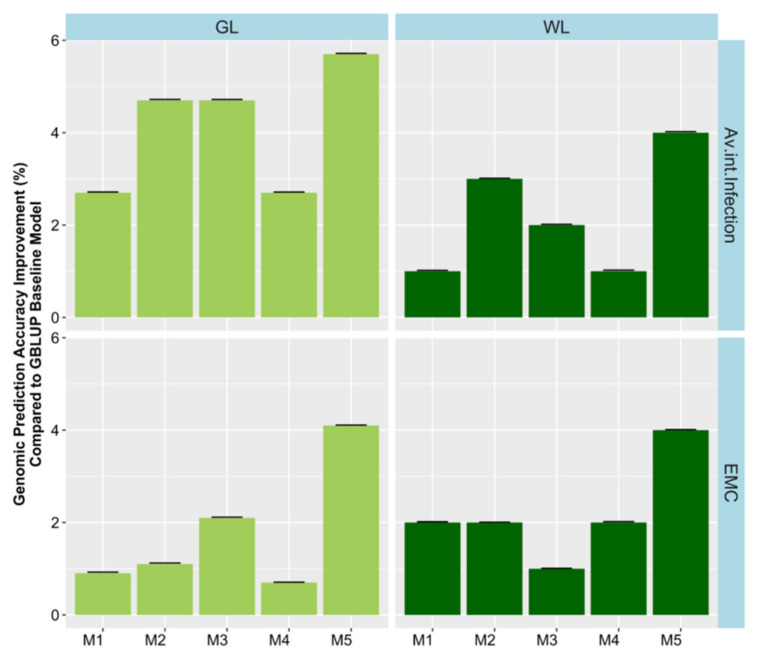
Genomic prediction accuracy improvement for each environmental factor combination across models (M1–M5) compared to the baseline GBLUP model. Models were: M1 = Adding fixed terms such as year, location and water into the GBLUP base model without interactions. In M2, M3 and M4 a random interaction term of genotype by year, location and water condition was added in each model, respectively. Finally, the fifth model M5 combined all random interactions. GL = Green Lake, WL = Wickliffe, and EMC = emergence count.

**Figure 8:**
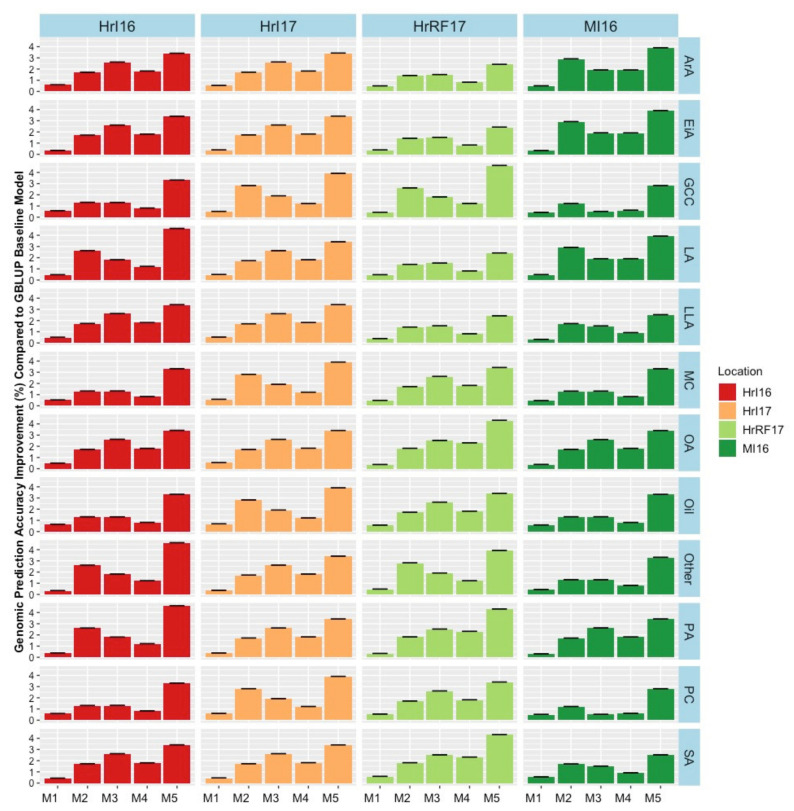
Genomic prediction accuracy improvement for quality traits for each environmental factor combinations across models (M1–M5) compared to the baseline GBLUP model for seed quality traits. Models were: M1 = Adding fixed terms such as year, location and water into the GBLUP base model without interactions. In M2, M3 and M4 a random interaction term of genotype by year, location and water condition was added in each model, respectively. Finally, the fifth model M5 combined all random interactions. Hr = Horsham, MI = Mininera, MC = moisture content, Oil = oil content, GCC = glucosinolate (μmol/g seed), PC = seed protein content, PA = palmitic acid (C16:0), SA = stearic acid (C18:0), OA = oleic acid (C18:1), LA = linoleic acid (C18:2), LLA = linolenic acid (C18:3), ArA = arachidic acid (C20:0), EiA = eicosenoic acid (20:1), and Other = other compounds.

The extent of G × E had not previously been investigated in this dataset in a genomic prediction context. Therefore, we extended the GBLUP models by incorporating random G × E interaction terms and investigated their effect on variance components and prediction accuracy. The environmental factor combinations investigated included year, location, and water condition (rain-fed or irrigated). G × E factors were investigated by adding them one at a time to the base model as well as with a full model containing all factors resulting in five different models. Substantial variances were found for interactions of genetics with environment (Table S4). However, the best fitting environmental interaction was not consistent across traits. In general, model 5, which fitted all interaction terms had the highest loglikelihood and lowest Akaiki information criterion (AIC, Table S4). Incorporating G × E factors in the model M_1_ to M_5_, improved the relative prediction accuracy across trials by a modest 0.5% to 6% for agronomic traits and 0.2% to 4% quality traits (Figure 6, Figure 7, and Figure 8). Only minor increases in accuracy were observed in EMC and DTM. Some variability in the benefit of modelling G × E interactions was observed across trials. Modelling rain-fed versus irrigated status tended to show the smallest increases, followed by location and year effects. Including all interactions simultaneously in the model resulted in the largest accuracy improvement. Our findings suggest that these gains could be potentially improved in the future as more data, including additional locations and years, is added, allowing more accurate distinction of the interactions from the baseline model.

## 3. Discussion

A total of 22 traits were investigated for genetic correlations and genomic prediction accuracies for agronomic, disease, and fatty acid traits using 202 canola (*Brassica napus* L.) lines genotyped with genotyping-by-sequencing (GBSt). Phenotypic and genetic correlations were calculated within and between all traits across locations. In particular, the correlations have shown a relationship of several FAs with blackleg disease resistance. In this study, we achieved moderate to high genomic prediction accuracy across most traits. The highest prediction accuracy was recorded for AvInf and YIELD. Below average prediction accuracies were observed for EMC and VIG. Extending the GBLUP model with G × E terms modestly increased prediction accuracies.

### 3.1. Heritability and Genetic Correlations

Broad-sense (H^2^) and narrow sense genomic (h^2^) heritabilities followed similar trends for agronomic traits and were similar of those found in previous studies [32,33]. Our moderate heritabilities for quality traits also confirmed previous published results [13,14,34].

The genetic correlations for agronomic and disease-resistance traits across years and locations were generally high. One would expect this unless G × E interactions explain a large proportion of the genetic variance. This pattern was confirmed a recent review by Oakey et al., 2016 [35] in crop multi-environment trials which found positive correlations between similar agronomic traits in different years. YIELD was positively correlated with PH, DTF, LOD, Shatter and SurvRt. Würschum et al. (2012) reported similar correlation trends in canola doubled haploid population We found that blackleg disease was positively correlated with shattering, which agrees with several published studies indicating the effect of blackleg disease is at the whole plant level including pods [36,37]. As expected, YIELD was strongly correlated with SurvRt, AvInf and PLH.

Improving the canola quality profile is of high priority in canola breeding. As result of this focus, canola Oil and PC has been increased, shelf life and frying quality were improved, and EiA and GCC were reduced. This has made canola more palatable and desirable for both human consumption and as animal feed [9]. Genetic correlations can help our understanding of the relationship between FA traits. Oil was positively correlated with ArA (C20:0), EiA (C20:1) and SA (C18:0) and negatively correlated with GCC, LA (C18:2) and LLA (C18:3). These relationships can be at least in part attributed to the underlying biosynthesis pathways [5,38]. For example, OA was positively correlated with EiA, LA and LLA and OA is known to be precursor in the pathways to these FAs [34,39,40,41].

The correlation structure of agronomic and quality traits has confirmed those reported in the literature. Oil was positively correlated with most of the agronomic traits such as YIELD, DTF, DTM, PH and SurvRt. A negative correlation was found between LA (C18:2) or LLA (C18:3) and seed yield suggesting that the content of these predominant FAs relatively decreases as YIELD increases. Our finding is in agreement with Gacek et al., 2016 [9], who found a similar trend between FA compositions and seed weight using 60 doubled haploid canola populations genotyped using 91,205 SNPs with a Skim genotyping-by-sequencing (GBS) method. LOD was negatively correlated with Oil, PC and other FAs, confirming Kendall et al. (2017), indicating the impact of LOD on profitability is not only through YIELD losses, but also through reductions in quality. We found that GCC was negatively correlated with YIELD, which is in line with Wurschum et al., 2012 [42]. The correlation pattern between agronomic and quality traits have been reported in a variety of oilseeds, such as sunflower [43], oat [44], soybean [45], and canola [6].

Fatty acids have many functions, of which only some are related to human health, such as OA’s cholesterol-lowering properties [46]. FAs also have structural molecules with functions involved in regulating and signalling which are invoked by environmental changes and development [47,48]. Of particular interest to us was the FA relationship to blackleg disease resistance, which we investigated with genetic correlations. We found that AvInf was strongly negatively correlated with ArA, SA, and strongly positively correlated with GCC. No precedent for this pattern was found in the literature for seed quality. Nevertheless, these results agreed with Cohen et al., 1991 [49], they reported that foliar application of ArA and EiA induced 94% and 97% protection against *Phytophthora infestans* in potato. Weaker positive relationships of AvInf were found with PA, LA, and LLA. This was in agreement with Wurschum et al., 2012 [42], which found that soybean seed infected with *Phomopsis* spp. was positively correlated with LA and LLA, and negatively correlated with OA levels. However, in contrast to our findings, a correlation trend reported in eggplant [50] found that an increase in palmetoleic acid production enhanced resistance to *Verticillium dahlia* disease. In the study of Xue et al., 2008 [51], fungal biomass of infected seed had a positive relationship with oleic:linoleic acid (O:L) ratios and oleic acid with the fungal pathogen *Cercospora kikuchii* in soybean. In our study, OA was slightly negatively correlated with AvInf.

Accounting for these relationships among all relevant traits is important during breeding. In particular, the strong relationships between some FAs and AvInf should be considered (once confirmed in other populations), albeit they seem mostly in the favourable direction of increased GCC and lower SA. Genomic selection allows the selection for all traits simultaneously and formulating a selection index that incorporates these genetic correlations and proper economic weights will be key to ensuring genetic progress for all key traits. It may also be possible to link the screening of FA to blackleg disease using advanced breeding tools.

### 3.2. Genomic Prediction with and without G × E Interactions

This was a first report evaluating genomic prediction accuracy in a wide range of agronomic traits, disease and FA traits under rain-fed and irrigated conditions in one study. We found a high prediction accuracy for seed yield, oil content and average internal infection at Mininera and Horsham irrigated 2016 and 2017. Delourme et al., 2006 [52] reported that phenotypic variation in oil content is controlled by a few major quantitative trait loci (QTL) in canola, which may make FA traits easier to predict. Lower accuracy was recorded for emergence count, lodging score and most of FAs. Overall, agronomic traits had higher prediction accuracy than FA traits within sites and year. A similar result by Wurschum et al., 2014 [13] in a winter doubled haploid population and Jan et al., 2016 [14] in spring lines showed that a higher prediction accuracy for yield, flowering time and plant height than for quality traits. FA profiles were predicted using near infrared spectroscopy (NIR). Some of the FAs are present in very small amounts in canola and NIR predictions of such FAs with NIR were thought to not be as accurate than with gas chromatography. Nevertheless, genomic prediction was able to predict all FAs with moderate accuracy in our study, which would confirm that the measurements even of FA <1% were sufficiently accurate. NIR prediction equations depend on the training data sets used and on methodology, so it is possible that other NIR predictions of <1% FAs are less accurate than ours and that genomic prediction would not be feasible in that case. It is strongly recommended to perform cross-validations to determine genomic prediction accuracy for all FAs before using them for selection.

Furthermore, we investigated the effect of incorporating (G × E) interactions in the GBLUP model. The aim was to investigate G × E in Australian growing conditions to create a set of baseline variance components and prediction accuracies that would inform future pre-breeding strategies. Including G × E interactions improved the model fit and interactions with watering protocol (rainfed or irrigated) and location explained more variance than the year interaction. Not unexpectedly, canola’s genetic response to water scarcity will be a key consideration going forward. In Australia, growing regions tend to be defined by the amount of rainfall they receive and target breeding environments will mirror these definitions. While our study had a least four environments for each trait, it was still somewhat limited in terms of inferences that can be drawn from its results (e.g., some interaction terms fixed at boundary, Table S4). Additional data from a more diverse set of environments is needed to draw more definite conclusions. Nevertheless, the inclusion of environmental interactions in genomic prediction improved prediction accuracy.

The inclusion of genotype by year, location, and water conditions (irrigation and rain-fed) improved prediction accuracy up to 6% compared to the standard GBLUP model. Our findings are in line with several studies that have demonstrated a benefit of including G × E terms in genomic selection [26]. We achieved high accuracies across environments, which was likely due to several factors. First, while in the blackleg traits a wider range of locations was tested, in the agronomic and seed quality traits there were only two locations and the main difference between trials was the watering regimen. Furthermore, we phenotyped the same lines across all trials. Should either the target environments or the reference population become more variable in future testing, we would expect a decrease in prediction accuracy due to increased G × E or decreased genetic connectedness, respectively.

Our G × E models will have to be expanded to include weather and soil information to make them more general and more applicable to breeding. Using a G × Year effect, while a useful academic exercise, cannot be applied in breeding as we do not know future years. Environmental covariates have also been included in several previous studies [27,29,53]. In disease-resistance traits, the forecasted presence or absence of blackleg races based on weather patterns, geography and in-field sampling would also be useful to include in predictions. Furthermore, more sophisticated G × E models, such as factor analytic models, have also been applied in a range of crops (e.g. [54]). In several cases, these approaches have been able to provide insight into clustering of sites and could potentially increase the accuracy of genomic prediction in our dataset.

## 4. Materials and Methods

### 4.1. Plant Material and Trial Designs

A total of 202 spring canola lines were used for this study. This set included historical Australian open pollinated and hybrid varieties (n = 71), advanced spring canola breeding lines (n = 70) and advanced germplasm from spring canola crossed with European winter canola (n = 63).

This spring canola set was evaluated by in-field phenotyping conducted at six experimental sites in Victoria, Australia. These sites were at Wickliffe (WL15) and Green Lake (GL15) in 2015; Mininera (MI16), Horsham irrigated (HrI16; HrI17) and Horsham rain-fed (Hr17) in 2016 and 2017. The irrigated trials had two water applications by flood irrigation, one in January to the equivalent of 100 mm of rain, and one in April to the equivalent of 30–40 mm of rain. These trials were no longer irrigated after sowing, receiving only the natural rain fall of the season. The 2015 trials were rain-fed disease nurseries for blackleg sown in the stubble from the previous year’s canola crop (ATR-Gem). The trial design was undertaken in AGROBASE using a randomized complete block design with a check variety grid (var. Trigold). The trials were sown in single rows (5 m long by 0.75 m apart) at 150 seeds per row, and two replications per site [15]. The same set of lines were sown in agronomic plot trials during the 2016 and 2017 under irrigated and rain-fed conditions. Lines were sown in 5 m long and 1.5 m wide plots in a randomized incomplete block design with 2 or 3 replications per location. The agronomical trials were protected against blackleg infection by applying in-farrow fungicide at sowing and foliar fungicide at the 6–8 leaf stage.

### 4.2. Phenotyping and Trait Measurements

A total of 22 traits were recorded across years and locations such as emergence counts (number of seedlings per row emerged 6 weeks after sowing) and adult plant survival count (number of plants per row at maturity) were recorded (Table S1). These were used to calculate the survival rate (SurvRt) as the ratio of the survival plant count at maturity to the emergence count. Average internal infection (AvInf) for blackleg was measured in all years as the area of the stem infected by the fungus *Laeptosphaeria maculans* recorded as the percentage of affected area of the total stem cross-section, as described in the Spring Blackleg Management Guide [37]. In the 2015 disease nurseries, to estimate AvInf, a maximum of 20 randomly selected plants per row were cut with secateurs at the crown and the cross section of the stem was examined for fungal growth [15]. In the 2016 and 2017 agronomic trials, the blackleg AvInf was determined from a maximum of 5 consecutive plants cut at the crown level and observed. Seedling emergence (EMC) and vigor (VIG) were recorded as a visual score from 1 to 9 (where 9 was the maximum, and 7 was adequate and 1 was very poor). Days to flowering (DTF) was defined as the number of days from sowing until 50% of plants had the first flower opened per plot. Days to maturity (DTM) was the number of days from sowing to physiological maturity. Lodging (LOD) was scored on a scale from 1 to 9 (where 1 was flat and 9 was upright). Plant height (PLH) was the above ground height of the plants in a plot, in centimetres. Seed yield per plot (YIELD) was recorded in g/plot and shattering (SHA) measured the amount of shattering on a 5 (completely shattered) to 0 (no shatter) scale.

Seed quality traits were measured at MI16, HrI16, HrI17 and Hr17 and determined by near infrared spectroscopy on whole seeds using the Foss XDS Rapid Content Analyser (Foss Denmark, Hillerod, Denmark) according to Golebiowski et al., 2005 [12]. The algorithms for each seed trait were developed using the peer reviewed protocols as described for each trait. Seed moisture content (MC), total oil (Oil, % volume per seed dry weight), oil content and protein were determined as described by the American Oil Chemists Society [55]. The algorithms were validated using the reference methodologies. Moisture content, total oil by solvent extraction using the Soxhlet apparatus, (Berlin, Germany), and total seed protein was determined by the Dumas combustion methods using the Leco TruSpec analyser (St. Joseph, MI, USA), American Oil Chemists Society [55].

Total seed glucosinolates (GCC; μmol/g seed) according to Mailer et al., 1978 [56]. Fatty acid (FA) composition PA = palmitic acid (PA, %), stearic acid (SA, %), Oleic acid (OA, %), linoleic acid (LA, %), linolenic acid (LLA, %), arachidic acid (ArA, %), eicosenoic acid (EiA, %) was determined using NIR as described by the American Oil Chemists Society [55] spectroscopy and based algorithms developed to determined fatty acids using gas liquid chromatography [57]. AOAC standards were used to validate each fatty acid.

Prior to genomic prediction analysis, phenotypes were adjusted for spatial variability with autocorrelation error (AR1 × AR1) models for field condition variability to generate Best Linear Unbiased Estimates (BLUEs) using ASReml [58] as described in Fikere et al., 2018 [15]. The following model was applied for line i sown at row j and column k,

$$y_{ijk}=\mu +g_{i}+r_{j}+c_{k}+e_{ijk}$$

where *y_ijk_* is the phenotype, µ is the overall mean, *g_i_* is the fixed effect for variety *i*, *r_j_* is the random effect for row *j*, *c_k_* is the random effect for column *_k_* fit as spline, and *e_ijk_* is the residual. Log likelihoods and uniformity of residual variogram plots were used to judge the best model. BLUE summary statistics for the whole population are presented in Table S2.

### 4.3. Genotype Data, Quality Control, and Imputation

A total of 202 spring lines were successfully genotyped with a transcriptome GBSt assay using the protocol described in Malmberg et al., 2018 [59]. The following quality control thresholds were applied: minor allele frequency <0.01, read depth <5, SNP heterozygosity <0.4, missing rate per SNP > 50% and monomorphic SNP were removed from the dataset. We used BEAGLE version 4.1 [60] to impute sporadic missing genotypes. SNP with Beagle Rsq < 0.5 were excluded, resulting in 62,082 SNPs for subsequent analysis (Figure S1). Note that chromosomes 1–10 were allocated to sub-genome A and chromosomes 11–19 to sub-genome C. Whole-genome sequence for 87 canola lines is available in NCBI Sequence Read Archive (PRJNA435647, [61]). The remaining genotype and phenotype data supporting the conclusions of the manuscript are available upon request from the authors, without undue reservation, to any qualified researcher.

### 4.4. Heritability, Genetic Correlations and Genomic Prediction

Broad sense (H^2^) and genomic heritability (h^2^) were estimated as described in Fikere et al., 2018 [15] using Restricted Maximum Likelihood (REML) in ASReml version 3 [58] as the ratio of the variance due to lines or the additive variance to the phenotypic variance [62]. Specifically, BLUEs were fitted as phenotypes and lines were fitted either as independent random effects for H^2^ or with the genomic best linear unbiased prediction (GBLUP) model described below for h^2^. For genomic models we utilised GBLUP:

(base model) $$y=1_{n}\mu +Xb+Zg+e$$

where **y** was the vector of BLUEs, **1**_n_ was a vector of the 1s, µ was the overall mean, **X** and **Z** were the design matrices, **b** was a vector of fixed effects, **g** was a vector of genomic breeding values $\sim N\left(0,G\sigma_{g}^{2}\right)$, and e was a vector of a residuals$\sim \;N\left(0,I\sigma_{e}^{2}\right)$, $\sigma_{g}^{2}$ was the genetic variance, and $\sigma_{e}^{2}\;$was the error variance. The genomic relationship matrix (**G**) was derived as in Yang et al., 2010 [63] Genomics allows the estimation of genetic correlations which were previously impossible as it increases line connectedness. However, in our case, all lines had been phenotyped in all trials. We calculated genetic correlations for all traits and trial combinations. In the bivariate GBLUP model, y was a 2n vector and the mixed model equations were expanded accordingly. In single trait GBLUP models (e.g., to calculate genomic heritability), **y** was a vector of n rows. Cross-validations to evaluate genomic prediction accuracy were also run with single trait models. The genetic variance was estimated with the REML GBLUP model and genomic heritability was calculated as ratio of genetic to phenotypic variance (corrected for replication). We assessed the effect of fitting G × E interaction terms within GBLUP.

The base model was extended to include environmental factors to allow for the combination of field trials across year, location, and watering (rain-fed or irrigated) factors to establish the extent of G × E in this dataset. Specifically, the G × E interactions were modeled as random **G** by environment factors. Five additional models were fitted, which investigated across factor prediction with G × E terms, first one factor interactions at a time and then all interactions simultaneously. All additional models fitted year, location, and watering as fixed effects.

(1) Model 1. y=1μ+Xb+Zg+e

was the baseline plus fixed effects year, location and water

(2) Model 2. y=1μ+Xb+Zg+Yg.y+e

Added a random effect **Yg** **y** for the **G** by year interaction

(3) Model 3. y=1μ+Xb+Zg+Lg.l+e

Added a random effect Lg.l for the G by location interaction

(4) Model 4. y=1μ+Xb+Zg+Wg.w+e

Added a random effect Wg.w for the G by water interaction\

(5) Model 5. y=1μ+Xb+Zg+Yg.y+Lg.l+Wg.w+e

Added all **G** by environment interactions

### 4.5. Cross-Validation for the Genomic Prediction

We used 5-fold cross-validation within trials where the entire dataset was randomly divided into five subsets. Four of the folds (80% of lines) were used as the reference set, and the remaining fifth fold (about 20%) was used as the validation set. The phenotypes of the validation set were masked and predicted using GBLUP. Similarly, 5-fold-cross validations were used in the G×E interaction models. The combined set of trials were used as the reference set to predict the sub-set of validation lines, whose entire trial’s phenotypes were masked. The same process was repeated for each trial and G×E model. The accuracy of GS was estimated as the correlation between BLUEs and GEBVs. Cross-validations were repeated five times and the mean prediction accuracy across 25 subsets (i.e., 5 cross-validations each 5 fold) was calculated per scenario.

## 5. Conclusions

Moderate to high genomic prediction accuracies were achieved with simple GBLUP models and inclusion of G × E interactions improved accuracies slightly. The genetic correlations calculated highlighted potential relationships between FA traits with blackleg disease resistance. The developed genomic predictions and genetic correlations are the building blocks to initiate pre-breeding simultaneously for several key traits using genomic selection indices.

## Supplementary Materials

The following are available online at https://www.mdpi.com/2223-7747/9/6/719/s1: Figure S1: Accuracy of imputation by chromosomes in *Brassica napus* L. genome using BEAGLE software, Figure S2: Correlations between agronomic and disease traits across locations and years, Figure S3: Correlations between seed quality traits across locations and years. Table S1: Traits measured across trials and years, Table S2a: Summary statistics and broad sense heritability (H^2^) for agronomic traits at Wickliffe (WL) and Green Lake (GL) sites during the 2015 growing season, Table S2b: Summary statistic and broad sense heritability for agronomic traits at Mininera and Horsham irrigated 2016, 2017 sites and Horsham rain-fed 2017, Table S2c: Summary statistics and broad sense heritability (H^2^) for quality traits at Mininera and Horsham sites 2016 and 2017 growing season, Table S3a: Genomic prediction accuracy for blackleg nursery sites at Wickliffe and Green Lake sites during 2015, Table S3b: Genomic prediction accuracy for agronomic traits during 2016, Table S3c: Genomic prediction accuracy for quality traits using GBLUP method, Table S3d: Genomic prediction and bias score for agronomic traits using GBLUP model at Horsham rain-fed and Horsham irrigated sites 2017, Table S4: Variance components, Loglikelihood and Akaike information criterion (AIC) for average internal infection, seed yield, and oil content (%) from phenotypic models combining all field trials, where Int are interactions.

## Abbreviations

BLUEs	best linear unbiased estimates
DH	doubled haploid;
G × E	genotype-by-environment
GBLUP	genomic best linear unbiased prediction
GBSt	transcriptome genotype-by-sequencing
GEBVs	genomic estimated breeding values
GS	genomic selection
MET	multi-environment trial
NIR	near infrared spectroscopy
QTL	quantitative trait loci
REML	restricted maximum likelihood
SNP	single nucleotide polymorphism
