# Supplementary material for: Genomic Prediction and Genetic Correlation of Agronomic, Blackleg Disease, and Seed Quality Traits in Canola (Brassica napus L.)
Source: Plants (Basel). 2020 Jun 5;9(6):719. doi: 10.3390/plants9060719 (PMC7356366; doi:10.3390/plants9060719)
Supplement: Supplementary file 1 [file plants-09-00719-s001.pdf]

Genomic Prediction and Genetic Correlation of Agronomic, Blackleg Disease, and Seed Quality Traits in Canola (*Brassica napus* L.)

Mulusew Fikere[1,2,3], Denise M. Barbulescu[4], M. Michelle Malmberg[1,2], Pankaj Maharjan[4], Phillip A. Salisbury[2,5], Surya Kant[4], Joe Panozzo[4], Sally Norton[4], German C. Spangenberg[2,1], Noel O.I. Cogan[2,1], Hans D. Daetwyler[2,1]

SUPPLEMENTARY TABLES and FIGURES

Table S1. Traits measured at each of the six trial sites across three years. GL = Green Lake 2015, WL = Wickliffe 2015, MI = Mininera, Hr = Horsham.

	Location					
**Traits**	**GL**	**WL**	**MI16**	**HrI16**	**Hr17**	**HrI17**
AvInf	✓	✓	✓	✓	✓	✓
SurvRt	✓	✓	-	-	-	-
DTF	-	-	✓	✓	✓	✓
DTM	-	-	✓	✓	✓	✓
EMC	✓	✓	✓	✓	✓	✓
LOD	-	-	✓	✓	✓	✓
PLH	-	-	✓	✓	✓	✓
VIG	-	-	✓	✓	✓	✓
SHA	-	-	-	-	✓	✓
YIELD	-	-	✓	✓	✓	✓
MC	-	-	✓	✓	✓	✓
Oil	-	-	✓	✓	✓	✓
GCC	-	-	✓	✓	✓	✓
PC	-	-	✓	✓	✓	✓
PA	-	-	✓	✓	✓	✓
SA	-	-	✓	✓	✓	✓
OA	-	-	✓	✓	✓	✓
LA	-	-	✓	✓	✓	✓
LLA	-	-	✓	✓	✓	✓
ArA	-	-	✓	✓	✓	✓
EiA	-	-	✓	✓	✓	✓
Other	-	-	✓	✓	✓	✓

Table S2a. Summary statistics and broad sense heritability (H^2) for agronomic traits at Wickliffe (WL) and Green Lake (GL) sites during the 2015 growing season

Locations	Trait	Mean	SD	H^2	SE
WL	EMC	27.60	12.37	0.35	0.12
	SurvRt	34.06	18.68	0.54	0.13
	AvInf	79.80	12.82	0.61	0.14
GL	EMC	12.99	3.46	0.32	0.14
	SurvRt	58.73	15.31	0.48	0.08
	AvInf	55.96	16.49	0.54	0.11

Table S2b. Summary statistic and broad sense heritability for agronomic traits at Mininera and Horsham irrigated 2016, 2017 sites and Horsham rain-fed 2017

Mininera 2016					Horsham irrigated 2016			
Traits	Mean	SD	H^2	SE	Mean	SD	H^2	SE
AvInf	19.02	4.34	0.69	0.13	6.94	4.37	0.71	0.14
DTF	112.38	3.89	0.64	0.12	109.26	5.34	0.68	0.15
DTM	207.38	6.99	0.62	0.01	188.50	1.64	0.61	0.11
EMC	5.22	0.31	0.45	0.16	6.39	0.59	0.57	0.13
LOD	5.89	0.23	0.41	0.14	6.32	0.62	0.59	0.13
PLH	112.10	14.63	0.55	0.07	133.05	18.32	0.55	0.14
VIG	5.94	0.73	0.56	0.13	5.98	0.80	0.59	0.11
YIELD	1015.84	131.57	0.66	0.12	2217.11	368.56	0.70	0.12
Horsham rain-fed 2017					Horsham irrigated 2017			
	Mean	SD	H^2	SE	Mean	SD	H^2	SE
AvInf	17.27	5.39	0.61	0.14	11.45	3.12	0.58	0.11
DTF	109.00	3.66	0.68	0.11	114.06	4.92	0.59	0.15
DTM	162.62	30.69	0.61	0.12	185.45	1.80	0.49	0.13
EMC	5.42	1.08	0.33	0.15	4.36	0.30	0.35	0.12
LOD	7.34	0.04	0.59	0.11	5.92	0.46	0.52	0.12
PLH	134.99	8.04	0.55	0.14	151.46	5.96	0.58	0.13
VIG	5.92	0.49	0.59	0.13	6.10	0.40	0.47	0.15
SHA	0.32	0.23	0.45	0.13	0.40	0.27	0.51	0.13
YIELD	1566.59	315.10	0.72	0.15	2803.44	260.20	0.68	0.14

AvInf= average internal infection, DTF = days to flowering, DTM = days to maturity, EMC = emergence score, LOD = lodging score, PLH = plant height (cm), SHA = shattering score, YIELD = seed weight per plot (g/plot), VIG = vigor (score)

Table S2c. Summary statistics and broad sense heritability (H^2) for quality traits at Mininera and Horsham sites 2016 and 2017 growing season

	Mininera 2016				Horsham irrigated 2016				Horsham rain-fed 2017				Horsham irrigated 2017			
Traits	Mean	SD	H^2	SE	Mean	SD	H^2	SE	Mean	SD	H^2	SE	Mean	SD	H^2	SE
MC	5.21	0.17	0.44	0.12	5.62	0.17	0.46	0.16	5.54	0.17	0.47	0.14	5.59	0.13	0.51	0.13
Oil	45.10	1.80	0.49	0.14	44.51	1.87	0. 48	0.14	43.20	2.09	0.52	0.11	42.91	1.47	0.45	0.13
GCC	4.79	1.45	0.59	0.17	5.04	1.81	0.67	0.12	8.50	2.47	0.58	0.15	6.97	1.98	0.63	0.11
PC	20.01	0.67	0.57	0.14	20.60	1.05	0.66	0.14	22.38	1.41	0.60	0.11	22.96	0.87	0.68	0.14
PA	4.31	0.10	0.49	0.13	4.42	0.13	0.48	0.16	4.35	0.12	0.47	0.12	4.38	0.11	0.45	0.11
SA	2.78	0.14	0.53	0.08	2.65	0.15	0.57	0.12	2.83	0.13	0.55	0.13	2.69	0.11	0.61	0.13
OA	60.37	1.53	0.65	0.07	61.12	2.24	0.70	0.14	61.02	2.04	0.67	0.09	60.32	1.92	0.70	0.16
LA	17.71	4.71	0.54	0.16	17.27	4.58	0.56	0.05	20.62	1.33	0.54	0.08	20.78	1.45	0.52	0.09
LLA	12.09	4.79	0.58	0.14	11.83	4.54	0.64	0.13	8.25	0.79	0.59	0.07	9.00	0.70	0.67	0.11
ArA	0.60	0.03	0.61	0.14	0.62	0.03	0.68	0.09	0.64	0.03	0.65	0.11	0.63	0.03	0.66	0.13
EiA	0.90	0.03	0.41	0.11	0.98	0.03	0.49	0.11	1.05	0.03	0.47	0.12	1.06	0.03	0.41	0.16
Other	1.24	0.04	0.48	0.16	1.31	0.03	0.47	0.12	1.24	0.04	0.48	0.13	1.27	0.03	0.39	0.15

MC = moisture content (%), Oil = Oil content (%), GCC = glucosinolate (GCC; µmol/g seed), PC = Seed protein content (%), PA = palmitic acid (C16:0), SA = Stearic acid (C18:0), OA = Oleic acid (C18:1), LA = Linoleic acid (C18:2), LLA = Linolenic acid (C18:3), ArA = Archidic acid (C20:0), EiA = eicosenoic acid (20:1), Other

Table S3a. Genomic prediction accuracy for blackleg nursery sites at Wickliffe and Green Lake sites during 2015

Location	Traits	Accuracy	Bias	SE
	EMC	0.311	1.034	0.015
WL	Survival Rate	0.557	1.082	0.020
	AvInf	0.600	1.121	0.019
	EMC	0.289	0.97	0.014
GL	Survival Rate	0.329	1.147	0.017
	AvInf	0.423	1.113	0.025

Table S3b. Genomic prediction accuracy for agronomic traits during 2016

	Horsham irrigated 2016			Mininera 2016		
Trait	Accuracy	SE	Bias	Accuracy	SE	Bias
AvInf	0.537	0.019	0.942	0.469	0.022	1.081
DTF	0.475	0.018	1.049	0.432	0.016	1.063
DTM	0.504	0.018	0.892	0.385	0.008	2.065
EMC	0.408	0.023	1.135	0.315	0.009	1.555
LOD	0.533	0.026	0.896	0.339	0.019	1.445
PLH	0.509	0.026	0.992	0.379	0.012	1.492
VIG	0.442	0.021	1.133	0.381	0.013	2.348
YIELD	0.697	0.017	1.028	0.512	0.019	1.144

Table S3c. Genomic prediction accuracy for quality traits using GBLUP method

	Mininera 2016			Horsham irrigated 2016			Horsham rain-fed 2017			Horsham irrigated 2017		
Traits	Accuracy	SE	Bias	Accuracy	SE	Bias	Accuracy	SE	Bias	Accuracy	SE	Bias
MC	0.444	0.029	1.002	0.512	0.016	1.002	0.472	0.024	1.011	0.554	0.020	1.026
Oil	0.575	0.019	1.004	0.638	0.022	0.970	0.551	0.014	1.013	0.641	0.026	0.997
GCC	0.411	0.026	0.837	0.565	0.029	1.013	0.433	0.023	0.844	0.513	0.033	1.077
PC	0.486	0.014	0.964	0.575	0.021	0.968	0.525	0.011	0.971	0.592	0.014	1.022
PA	0.302	0.008	1.184	0.359	0.027	0.966	0.326	0.004	1.274	0.366	0.020	1.056
SA	0.540	0.019	1.065	0.415	0.027	0.877	0.571	0.015	1.145	0.435	0.018	0.961
OA	0.353	0.019	1.057	0.488	0.036	0.976	0.343	0.015	1.137	0.521	0.027	1.046
LLA	0.306	0.027	1.006	0.480	0.022	0.959	0.379	0.023	1.007	0.493	0.013	0.987
LA	0.451	0.026	1.113	0.452	0.017	0.935	0.463	0.022	1.104	0.466	0.016	1.175
ArA	0.478	0.025	1.134	0.582	0.019	1.059	0.473	0.021	1.054	0.515	0.018	1.193
EiA	0.318	0.013	1.432	0.343	0.027	1.137	0.369	0.009	1.342	0.366	0.021	1.161
Other	0.429	0.021	1.121	0.322	0.027	0.838	0.442	0.017	1.192	0.338	0.036	0.918

MC = moisture content (%), Oil = oil content (%), GCC = glucosinolates content ($\mu mol/g$), PC = seed protein content (%), PA = palmitic acid (C16:0), SA = Stearic acid (C18:0), OA = Oleic acid (C18:1), LA = linoleic acid (C18:2), LLA = linolenic acid (C18:3), ArA = arachidic acid (C20:0), EiA = eicosenoic acid (20:1), Other

Table S3d. Genomic prediction and bias score for agronomic traits using GBLUP model at Horsham rain-fed and Horsham irrigated sites 2017

	Horsham Rain-fed 2017			Horsham irrigated 2017		
	Accuracy	SE	Bias	Accuracy	SE	Bias
EMC	0.411	0.024	1.058	0.375	0.011	1.084
AvInf	0.507	0.022	1.047	0.577	0.018	1.005
DTF	0.402	0.031	1.511	0.493	0.024	1.043
DTM	0.345	0.024	1.305	0.442	0.025	0.893
PLH	0.516	0.021	0.981	0.475	0.017	1.066
LOD	0.341	0.013	1.062	0.491	0.019	1.041
SHA	0.367	0.018	1.144	0.351	0.025	1.356
VIG	0.309	0.011	2.277	0.334	0.019	1.468
YIELD	0.614	0.022	1.092	0.483	0.021	1.065

Table S4. Variance components, Loglikelihood and Akaike information criterion (AIC) for average internal infection, seed yield, and oil content (%) from phenotypic models combining all field trials, where Int are interactions. Five models as described in methods but without fitting genomic information, i.e. genetic variance is variance due to lines.

**Average Internal Infection**					
**Model**	**M1**	**M2**	**M3**	**M4**	**M5**
Genetic	23.39	34.18	18.04	19.07	28.2
GeneticSiteInt	NA	69.07	NA	NA	5.2
GeneticYearInt	NA	NA	26.76	NA	0.49
GeneticWaterInt	NA	NA	NA	5.11	72.05
Residual	62.71	11.9	41.31	60.68	8.04
**No random effects**	2	3	3	3	5
**log**	-3232.6	-3084.28	-3189.78	-3230.67	-3072.78
**AIC**	6469.1	6174.55	6385.55	6467.34	6155.56
**YIELD**					
Genetic	38372.4	26540.3	38372.4	38372.3	26540.4
GeneticSiteInt	NA	11727.6	NA	NA	11727.5
GeneticYearInt	NA	NA	0.004*	NA	0.004*
GeneticWaterInt	NA	NA	NA	0.01*	0.004*
Residual	40135.1	35881.1	40135.1	40135.1	35881.1
**No random effects**	2	3	3	3	5
**log**	-4831.97	-4824.87	-4831.97	-4831.97	-4824.87
**AIC**	9667.95	9655.74	9669.95	9669.95	9659.74
**Oil Content (%)**					
Genetic	2.29	2.29	2.22	2.29	2.22
GeneticSiteInt	NA	0*	NA	NA	0*
GeneticYearInt	NA	NA	0.201139	NA	0.2
GeneticWaterInt	NA	NA	NA	0*	0*
Residual	1.18	1.18	1.0455	NA	1.05
**No random effects**	2	3	3	3	5
**log**	-695.22	-695.218	-691.55	-695.218	-691.55
**AIC**	1394.44		1389.09	1396.44	1393.09

*values fixed at boundary

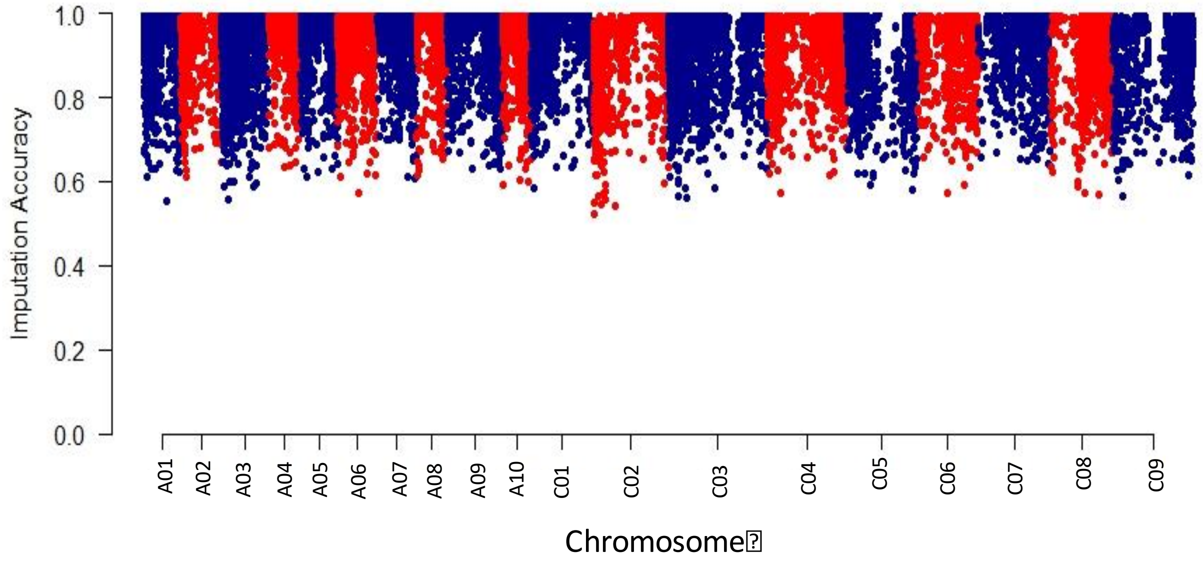

Figure S1. Accuracy of imputation by chromosomes in Brassica napus L. genome using BEAGLE software (Browning and Browning, 2007), after removing SNP markers below 50% imputation accuracy, giving a final dataset of 62,082 SNPs.

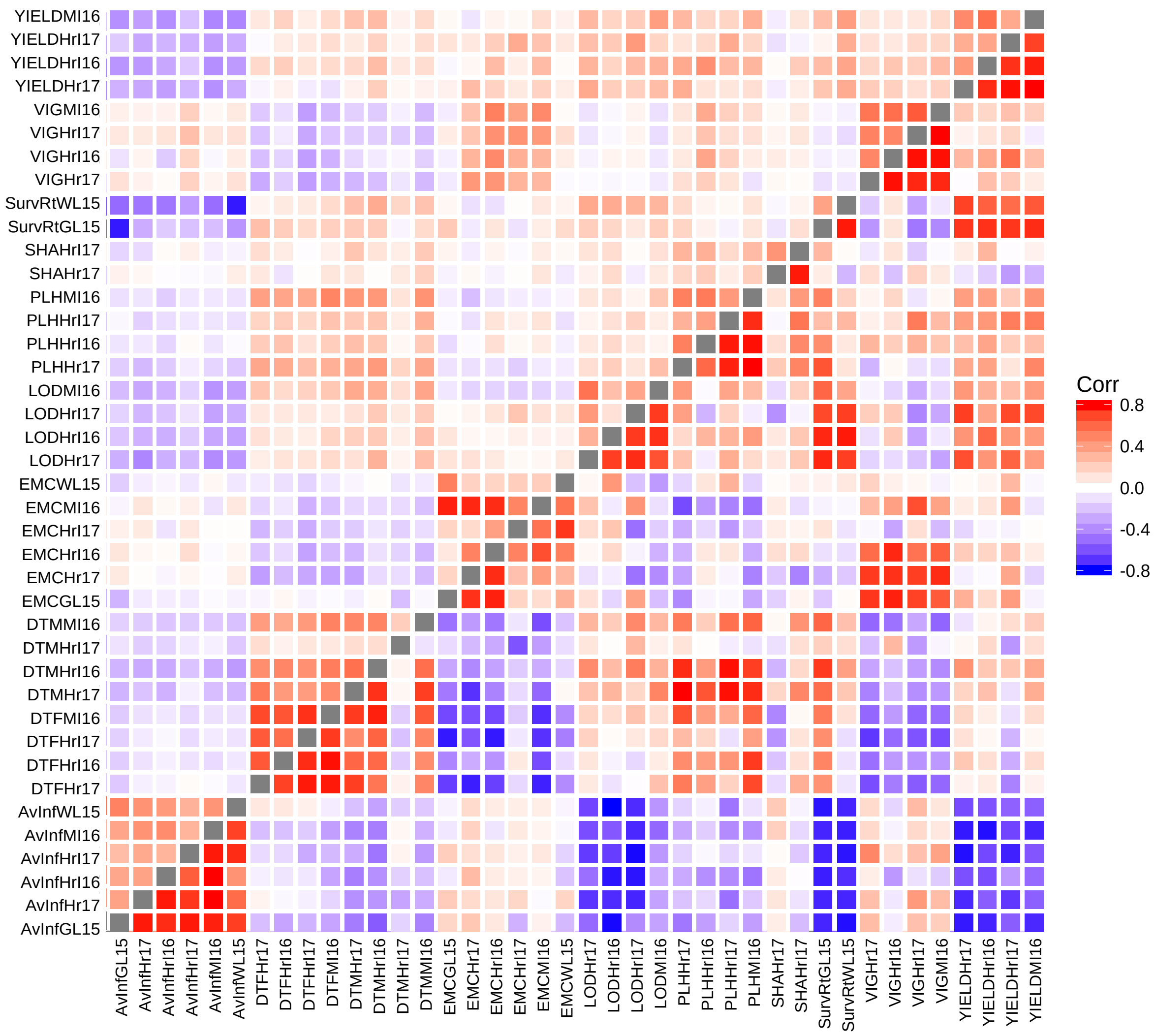

Figure S2. Phenotypic (above diagonal) and genetic (below diagonal) correlations between agronomic and disease traits across locations and years. Abbreviations: WL15 = Wickliffe 2015, GL15 = Green Lake 2015, MI16 = Mininera 2016, HrI16 = Horsham irrigated 2016, HrI17 = Horsham irrigated 2017, Hr17 = Horsham rain-fed 2017 sites. AvInf = average internal infection, SurvRt= survival rate, DTF = days to flowering, DTM = days to maturity, EMC = emergence count, LOD = lodging percentage, PLH = plant height (cm), SHA = shattering, YIELD = seed weight per plot, VIG = vigor. Mean, min, and max of genetic correlations SE 0.26, 0.04, 0.9, respectively. Mean, min, and max of phenotypic correlations SE 0.08, 0.02, 0.08, respectively.

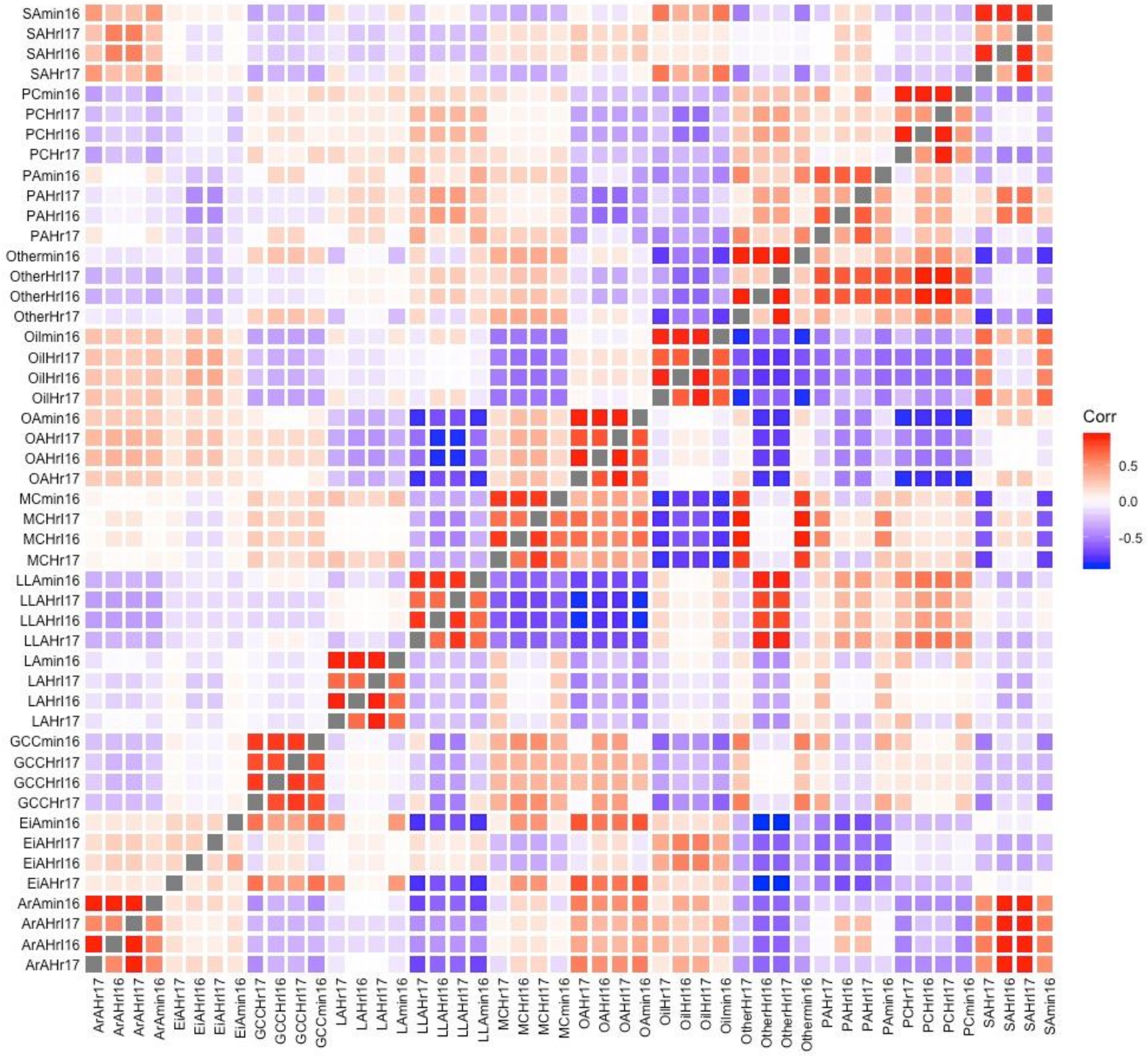

Figure S3. Phenotypic (above diagonal) and genetic (below diagonal) correlation between seed quality traits at Horsham irrigated (HrI 2016, 2017 and Horsham rain-fed, Hr 2017 and Mininera sites during the 2016 growing season. MC = moisture content (%), Oil = oil content, GCC = glucosinolate (μmol/g seed), PC = seed protein content, PA (C16:0) = palmitic acid, SA (C18:0) = stearic acid, OA = oleic acid (C18:1), LA = linoleic acid (C18:2), LLA = linolenic acid (C18:3), ArA = arachidic acid (%, C20:0), EiA = eicosenoic acid (C20:1), Other. Mean, min, and max of genetic correlation SE 0.26, 0.04, 0.9, respectively. Mean, min, and max of phenotypic correlations SE 0.08, 0.02, 0.08, respectively.
